# Microsurgical Treatment of a Giant Intracavernous Carotid Artery Aneurysm in a Pediatric Patient: Case Report and Literature Review

**DOI:** 10.7759/cureus.34010

**Published:** 2023-01-20

**Authors:** Edgar Nathal, Javier Degollado-García, Alfredo Bonilla-Suastegui, Héctor A Rodríguez-Rubio, Bill Roy Ferrufino-Mejia, Martin Roberto Casas-Martínez

**Affiliations:** 1 Neurosurgery, Instituto Nacional de Neurología y Neurocirugía Manuel Velasco Suárez, Mexico City, MEX; 2 Neurosurgery, Instituto Nacional de Neurología y Neurocirugía Manuel Velasco Suárez, Mexico city, MEX

**Keywords:** intracavernous carotid artery aneurysm, cavernous sinus aneurysm, pediatric neurosurgery, vascular neurosurgery, trapping, bypass

## Abstract

Intracranial aneurysms in children account for 4%-5% of all cases, with 20% being considered giant (>25 mm). The main sites of occurrence are the internal carotid artery (ICA) and the middle cerebral artery (MCA). Rupture and secondary subarachnoid hemorrhage occur in approximately 55%-72.5% of cases, with a 10%-23% mortality rate. We report the case of a previously healthy nine-year-old girl who developed sudden, severe right retroocular pain and a holocranial headache as a mode of onset. Besides, the patient presented with double vision, and her relatives sought medical attention. Paresis of the right III, IV, and VI cranial nerves was found at physical examination. An MRI and digital subtraction angiography showed the presence of a giant aneurysm in the cavernous portion of the ICA with a mass effect. The patient was treated surgically through a high-flow bypass using a radial artery graft and trapping of the aneurysm. She had an uneventful postoperative course and was discharged three days after the operation to continue follow-up at the outpatient clinic. The therapeutic options were: a) an endovascular approach using flow diverters or stenting and coiling; or b) surgical treatment with proximal closure of the ICA if the patient had good collateral circulation or trapping the aneurysm combined with a high-flow bypass if the collateral circulation was not good or absent. After discussion, we decided on the surgical option. Even when the surgery was successful in this case, there is no consensus about the best way to treat it; the selection should be based on the center´s experience when confronting this rare entity.

## Introduction

Pediatric intracranial aneurysms account for 4%-5% of all cases and have an annual incidence rate of 0.7% [1, 2, 3]. Aneurysms of the anterior circulation represent 75% of the cases. The main sites of occurrence are the internal carotid artery (ICA) and the middle cerebral artery (MCA), representing 27% and 26% of the total, respectively. In the posterior circulation, the basilar top is the most common site of location [3]. About 55%-72.5% of cases present rupture and secondary subarachnoid hemorrhage (SAH), with a 10%-23% mortality [1,2,3]. Approximately 27.5%-30% of unruptured aneurysms present with thunderclap headache without SAH, partial III nerve palsy, ischemic stroke, and seizures [2]. Saccular aneurysms exceeding 25 mm in maximal diameter have traditionally been classified as "giant" in size. Giant lesions comprise approximately 2%-5% of all cases. Still, in children, they occur with a far greater frequency (about 20% in some reported series). Approximately 40% occur in the carotid distribution, 25% in the anterior and middle cerebral arteries, and 30% in the vertebrobasilar territory [4]. In this report, we discuss the case of an unruptured giant intracavernous carotid artery aneurysm presenting as acute cavernous sinus syndrome in a previously healthy nine-year-old female patient. The surgical strategy used to solve the case is also discussed.

## Case presentation

**Figure 1:**
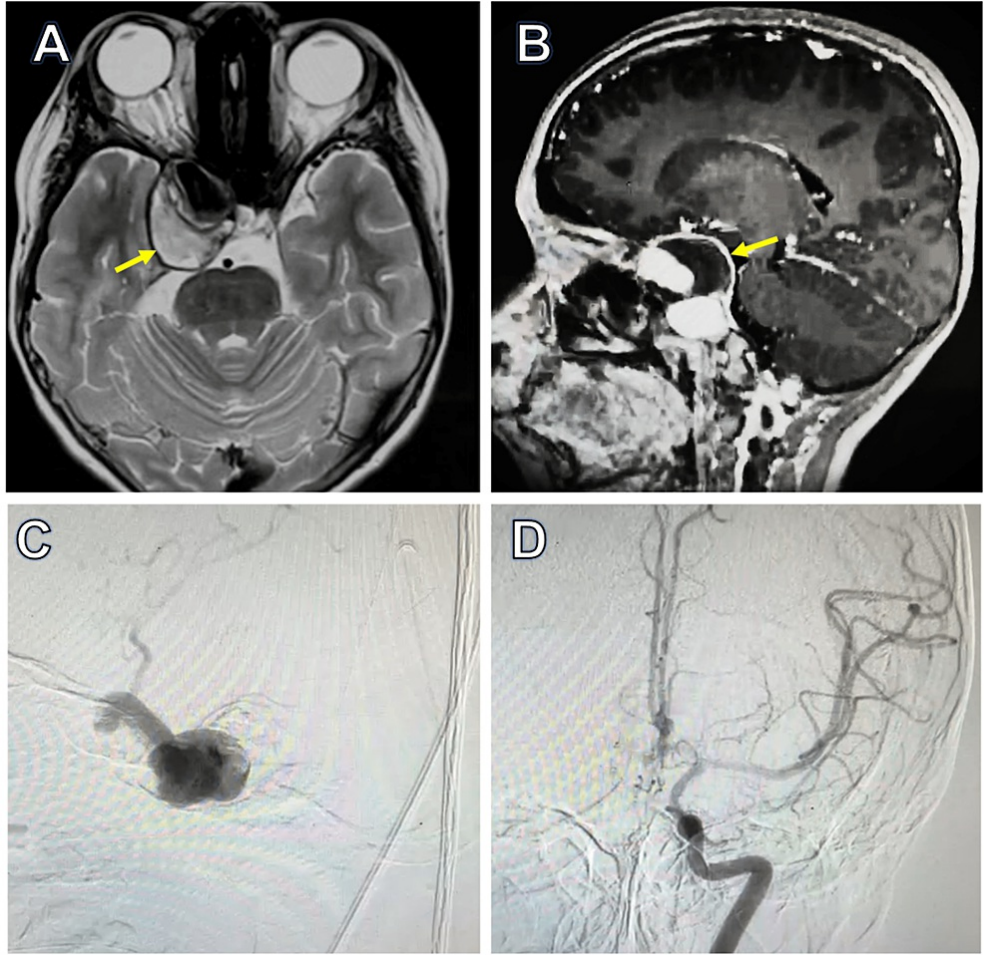
Preoperative scans. MRI axial (A) and sagittal views (B) show an aneurysm with partial thrombosis of 54 mm in diameter in the cavernous segment of the right ICA (arrows). The angiography images (C) and (D) confirmed the presence of the aneurysm with poor collateral circulation. MRI: magnetic resonance imaging, ICA: internal carotid artery

A nine-year-old girl with no significant medical history presented with pulsatile right retroocular pain and secondary holocranial headache, both of which were accompanied by nausea and vomiting. She received medical attention and was diagnosed with ocular infection. Three days later, her mother observed the absence of ocular movements, and the patient was sent to an ophthalmologist, who diagnosed probable right orbital cellulitis. The patient was redirected to a pediatric third-level hospital. The neurological examination showed a right palpebral ptosis, a mydriatic pupil that was not reactive to light stimuli, and complete paralysis of the right III, IV, and VI cranial nerves. An MRI study revealed a giant aneurysm in the cavernous portion of the right ICA (Figure 1).

Angiography with a balloon occlusion test (BOT) was completed and showed poor collateral circulation. After analysis of the case, the vascular neurosurgery department decided to perform trapping of the aneurysm with a high-flow bypass from the external carotid artery to the middle cerebral artery with a radial artery graft.

Surgical technique

**Figure 2:**
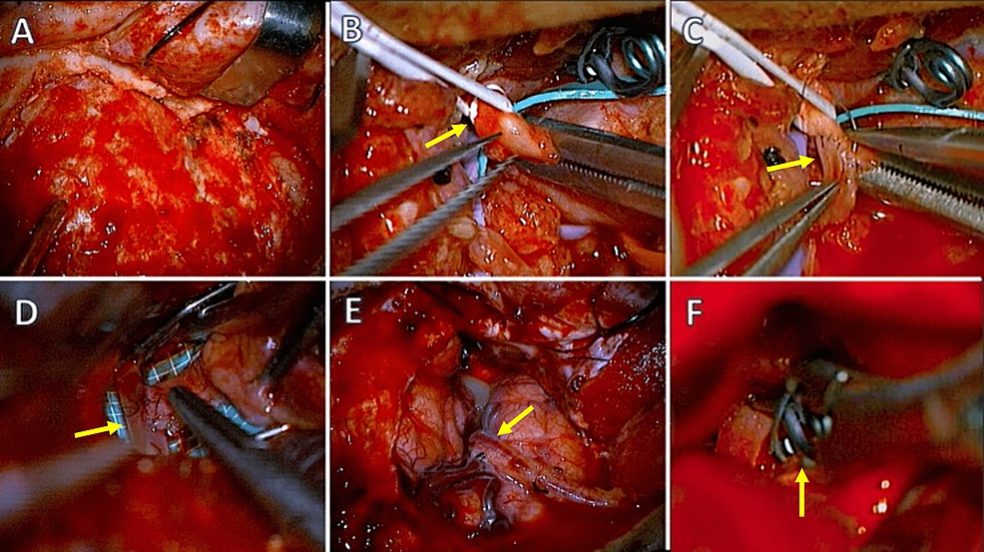
Intraoperative images. (A) right pterional craniotomy; (B) the carotid bifurcation is identified, and the external carotid artery is isolated (arrow); (C) a termino-lateral anastomosis is made with a radial artery graft previously obtained from the left arm with an 8-0 suture. After completion of the anastomosis, the clamp is released (arrow), and patency of the proximal bypass is confirmed; (D) the recipient artery is prepared at the level of the Sylvian fissure. The inferior trunk of the MCA is selected for the second end-to-side anastomosis. The anastomosis is completed with a 9-0 suture using separate points (arrow); (E) the anastomosis is completed (arrow), and its functioning is verified with the intraoperative Doppler; (F) after closing the ICA at the bifurcation with a suture, a clip is positioned just proximal to the origin of the ophthalmic artery to trap the cavernous sinus aneurysm permanently (arrow). MCA: middle cerebral artery; ICA: internal carotid artery

The patient was positioned supine with a left-side head rotation of 30° and fixed with the three-pin Mayfield head holder. A pterional approach was completed with an additional neck incision following the anterior border of the sternocleidomastoid muscle for carotid exposure. The carotid bifurcation was identified and isolated. Simultaneously, the left forearm was prepared to receive a radial artery graft. A previous Allen test showed good collateral blood flow at the palmar arch. After getting the graft, it was tunneled from the neck to the temporal side of the craniotomy. The inferior trunk of the M2 segment of the MCA was isolated with a dam. We completed a proximal end-to-side anastomosis between the radial artery graft and the external carotid artery using an 8-0 suture. After that, an end-to-side anastomosis between the radial graft and the middle cerebral artery was performed using nylon 9-0 (Figure 2).

**Figure 3:**
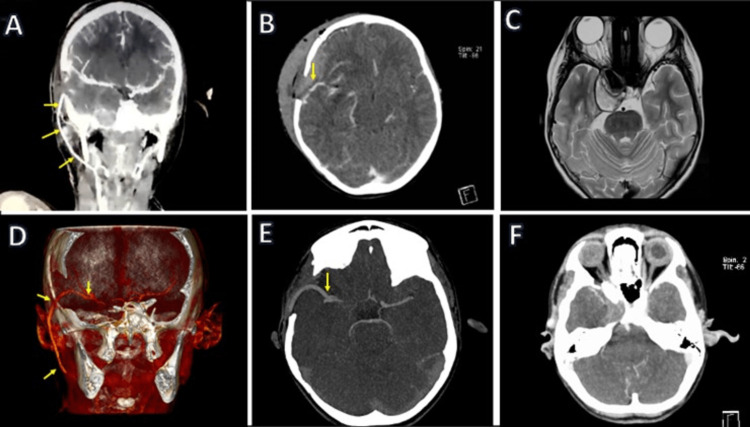
Postoperative images at two months (A–C) and six months (D–F) after surgery. Images (A) and (D) from the coronal view show a patent bypass (arrows). Images (B) and (E) show the CTA in axial view. The MCA's bypass (arrow) and branches are seen without evidence of low-density areas. Images (C) and (F) show pre- and postoperative (six months) images of the aneurysm. The mass effect is reduced with the improvement of the cavernous sinus symptomatology. CTA: computed tomography angiography; MCA: middle cerebral artery

The patency of the bypass was confirmed with fluorescein video angiography (FL-VAG) and intraoperative Doppler. The postoperative course was uneventful, and the patient was discharged three days later to continue with a follow-up at the outpatient clinic (Figure 3).

## Discussion

Clinical presentation

Treatment of giant cavernous aneurysms is still a matter of concern. There exists the possibility of endovascular treatment using flow diverters or some alternative technique such as stenting and coils ("jailing") [3,5,6]. However, some concerns exist about lifelong anti-platelet therapy, and the availability of these devices is limited in some countries. In the case of the "jailing" technique, there is the possibility of perpetuating the mass effect and producing permanent pressure over the cranial nerves [7]. Conversely, the surgical technique depends on the Bachelor of Occupational Therapy (BOT) results to demonstrate the grade of collateral blood flow. If the patient shows excellent flow, the simple proximal ligation of the ICA (Hunterian ligation) is enough to induce aneurysm thrombosis. In this case, some authors recommend completing a low-flow superficial temporal artery to middle cerebral artery (STA-MCA) bypass to avoid the occurrence of aneurysms in the long term on the contralateral side. In cases of insufficient collateral blood flow, trapping the aneurysm together with a high-flow bypass will solve the situation [3,8,9,10]. Our patient presented with cavernous sinus syndrome secondary to a giant aneurysm in the cavernous portion of the ICA. A similar case was reported in an 11-year-old child who presented with right eye exotropia associated with a severe headache [11]. In another case, the aneurysm caused severe retroocular pain, diagnosed as Tolosa-Hunt syndrome [12]. These syndromes are explained by the mass effect caused by the aneurysm over the lateral wall of the cavernous sinus.

Association with other pathologies

Some studies have described the relationship of intracranial aneurysms in children with diverse diseases such as problems in reticular fiber formation, gene mutations, connective tissue diseases (v. gr. Marfan’s syndrome, Ehlers-Danlos syndrome), coarctation of the aorta, fibromuscular dysplasia, sickle cell anemia, Paget syndrome, and tuberous sclerosis [2,3,8,13]. Approximately 15% of the cases can be attributed to an infectious etiology [11]. Further work did not show any of these conditions in our patient.

Treatment of choice

Regarding the treatment of choice, we conducted a bibliographic search in PubMed and Medline (EBSCO) about giant aneurysms of the cavernous sinus in the pediatric population (Table 1).

**Table 1:** Cases of giant intracavernous aneurysms in children have been published. NI: no information; Rt: right-side; Lt: left-side; Bil: bilateral; F: female gender; M: male gender; Mo: months; yrs: years

Case No	Authors	Year	Age/ Sex	Associated pathology	Clinical presentation	No.	Side	Treatment	Complications	Follow-up
											1	Devadiga et al. [14]	1969	1/M	None	Paresis of the left side	1	Lt	None	Dead	None
2	Banna et al. [15]	1990	1/F	Chest infection	Proptosis	1	Rt	Embolization	None	4 Mo
3	Linskey et al. [16]	1991	15/M	None	Orbital pain	1	Lt	Proximal occlusion	None	2 Mo
4	Cekirge et al. [5]	1996	4/F	None	Left third nerve palsy	2	Bil	Coiling	None	3 Mo
5	Sanai et al. [9]	2010	15/M	None	Diplopia and headache	1	Lt	Proximal balloon occlusion	None	1 Mo
6	Meyer et al. [10]	1989	NI	None	Mass effect	1	Rt	Proximal occlusion and bypass	None	2 yrs
7	Rehman et al. [8]	2010	11/M	Juvenile Paget’s disease	Left abducens nerve palsy	2	Lt	Proximal occlusion and bypass	None	2 Mo
8	Kumaria et al. [6]	2018	12/M	Septic arthritis	Diplopia	1	Lt	Flow diverter stent	None	3 yrs
9	Buño et al. [11]	2018	11/M	Horner syndrome	Diplopia	1	Rt	Clipping	None	6 Mo
10	Present case	2022	9/F	None	Proptosis	1	Rt	Bypass and trapping	None	2 Mo

Intracranial aneurysms in the pediatric population are uncommon but have a higher risk of rupture than in the adult population [4]. In the case of cavernous sinus aneurysms, they present with a mass effect, acting like a tumor. In the case of rupture, they produce a carotid-cavernous fistula with ipsilateral ocular signs and symptoms. As was said before, treatment could be accomplished through clipping or endovascular coiling; however, for aneurysms at this location, studies show better results with clipping in terms of durability, recurrence rate, and less need for additional procedures [3,8]. Some authors, like Sania et al., describe a higher recurrence rate in endovascular therapy and a higher rate of aneurysm formation in children [9].

Regarding the endovascular treatment for a similar case like ours, there are reports of successful treatment of a giant aneurysm in a child using a flow diverter stent with a good evolution three years after the procedure [6]. On the other side, surgical treatment was used in a child with a bilateral giant aneurysm who underwent a bilateral STA-MCA bypass, followed by endovascular carotid artery occlusion with a balloon on both sides, tolerating both procedures without postoperative complications [8].

The outcome of giant aneurysms in the pediatric population

**Figure 4:**
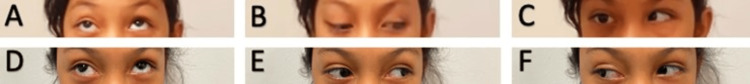
Postoperative evolution. Superior Row (A-C) shows the postop course at two months. (A) Partial opening of the right eyelid with adequate superior rectus muscle response and spontaneous pupillary reflex; (B) adequate superior oblique muscle response resolution of the right IV nerve lesion; and (C) paralysis of the right abducens muscle (VI nerve palsy) is still present. The inferior row (D-F) shows a postop course at six months. Images (D–E) show adequate function of the III nerve; image (F) shows complete remission of the right VI nerve palsy.

A retrospective multicentric study documented unfavorable outcomes with aneurysms larger than 5 mm, and this size was also independently associated with aneurysm recurrence. The annual aneurysm frequency, based mainly on 3 Tesla MRIs, was 0.7% [2]. In the case of cavernous sinus aneurysms, it is expected that the mass effect will improve after treatment, reducing the compression of the aneurysm over the cavernous sinus and alleviating the patient's symptoms. In our case, the extraocular motility deficits and proptosis resolved after surgical treatment, as had been previously reported in similar cases (Figure 4) [7,15].

## Conclusions

There has yet to be agreement on the best treatment for giant cavernous aneurysms in children. Favorable results depend on comorbidities, clinical presentation, and the centers' experience treating this rare entity, so the published cases still need to permit us to draw a unique conclusion. Regarding the treatment used in this case, we can say that proximal carotid occlusion, based on a BOT showing excellent collateral circulation, is an acceptable method of treatment to induce thrombosis of the aneurysm and further improve the symptoms caused by the mass effect. In cases of poor collateral circulation, a high-flow bypass and trapping the aneurysm are very good options for a permanent cure. Direct clipping of cavernous sinus aneurysms has been abandoned because of the high incidence of cranial neuropathies.
